# IDH2 Deficiency Aggravates Fructose-Induced NAFLD by Modulating Hepatic Fatty Acid Metabolism and Activating Inflammatory Signaling in Female Mice

**DOI:** 10.3390/nu10060679

**Published:** 2018-05-27

**Authors:** Jeong Hoon Pan, Hoe-Sung Kim, Kaleigh Elizabeth Beane, Allison Michelle Montalbano, Jin Hyup Lee, Young Jun Kim, Jun Ho Kim, Byungwhi Caleb Kong, Sangyub Kim, Jeen-Woo Park, Eui-Cheol Shin, Jae Kyeom Kim

**Affiliations:** 1 School of Human Environmental Sciences, University of Arkansas, Fayetteville, AR 72701, USA; jhpan@uark.edu (J.H.P.); kebeane@email.uark.edu (K.E.B.); ammontal@email.uark.edu (A.M.M.); 2 Department of Food Science, Gyeongnam National University of Science and Technology, Jinju 52725, Korea; eshin@gntech.ac.kr; 3 Department of Food and Biotechnology, Korea University, Sejong 30019, Korea; jinhyuplee@korea.ac.kr (J.H.L.); yk46@korea.ac.kr (Y.J.K.); jhkim@anu.ac.kr (J.H.K.); 4 Poultry Science, University of Arkansas, Fayetteville, AR 72701, USA; bkong@uark.edu; 5 Department of Pharmacology, Penn State University, Hershey, PA 17033, USA; kkojangnara@gmail.com; 6 School of Life Sciences and Biotechnology, BK21 Plus KNU Creative Bio-Research Group, College of Natural Sciences, Kyungpook National University, Daegu 41566, Korea

**Keywords:** IDH2, fructose, NAFLD, NF-κB, female mice

## Abstract

Fructose is a strong risk factor for non-alcoholic fatty liver disease (NAFLD), resulting from the disruption of redox systems by excessive reactive oxygen species production in the liver cells. Of note, recent epidemiological studies indicated that women are more prone to developing metabolic syndrome in response to fructose-sweetened beverages. Hence, we examined whether disruption of the redox system through a deletion of NADPH supplying mitochondrial enzyme, NADP^+^-dependent isocitrate dehydrogenase (IDH2), exacerbates fructose-induced NAFLD conditions in C57BL/6 female mice. Wild-type (WT) and IDH2 knockout (KO) mice were treated with either water or 34% fructose water over six weeks. NAFLD phenotypes and key proteins and mRNAs involved in the inflammatory pathway (e.g., NF-κB p65 and IL-1β) were assessed. Hepatic lipid accumulation was significantly increased in IDH2 KO mice fed fructose compared to the WT counterpart. Neutrophil infiltration was observed only in IDH2 KO mice fed fructose. Furthermore, phosphorylation of NF-κB p65 and expression of IL-1β was remarkably upregulated in IDH2 KO mice fed fructose, and expression of IκBα was decreased by fructose treatment in both WT and IDH2 KO groups. For the first time, we report our novel findings that IDH2 KO female mice may be more susceptible to fructose-induced NAFLD and the associated inflammatory response, suggesting a mechanistic role of IDH2 in metabolic diseases.

## 1. Introduction

Sugar-sweetened beverages, mainly consisting of high-fructose corn syrup (HFCS), are among the most popular refreshments in much of the world. Multiple studies have identified fructose as the major culprit of many diverse metabolic syndromes including non-alcoholic fatty liver disease (NAFLD) [1]. The consumption of fructose, largely in the form of HFCS, has been found to be particularly associated with negative metabolic outcomes such as triglyceride deposition in the liver, which is one of the hallmarks of NAFLD. In addition, increasing evidence suggests that fructose-induced metabolic syndrome is closely associated with chronic inflammation through inflammation signaling activation in the liver [2]. It is reported that intracellular reactive oxygen species production induced by a fructose-enriched diet plays a central role in the pathogenesis of fructose-induced liver disease [3]. In that regard, NADPH is critical because oxidized glutathione (i.e., endogenous cellular antioxidant) is regenerated via NADPH-consuming glutathione reductase and peroxidase systems [4].

Mitochondrial NADP^+^-dependent IDH (IDH2) catalyzes oxidative decarboxylation of isocitrate into α-ketoglutarate in the TCA cycle with concurrent reduction of NADP^+^ to NADPH [5]. Hence, IDH2 acts not only as a metabolic regulatory enzyme of the TCA cycle, but also as a major redox regulatory enzyme that prevents oxidative stress by producing NADPH. We previously reported that IDH2 deficiency influenced high-fat-diet-induced obesity [6] and hepatic steatosis [7] in aged male mice; yet it is not known how IDH2 deficiency is interlinked with NAFLD. Furthermore, a recent human study suggested that women are more prone to develop metabolic syndromes in response to fructose-sweetened beverages [8]. Therefore, in this preliminary study, wild-type (WT) and IDH2 knockout (KO) female mice were fed with fructose-supplemented water to investigate the effects of IDH2 deficiency on fructose-induced dysregulation of hepatic inflammatory signaling.

## 2. Materials and Methods

### 2.1. Animal Housing Conditions

Mice (four weeks old; female) were randomly assigned to experimental groups and then acclimated for the first seven days prior to fructose intervention. Commercial pelleted diet (AIN-76A; Central Lab Animals Inc., Seoul, Republic of Korea) and tap water were provided ad libitum during this acclimation period. Temperature (23 ± 2 °C), humidity (50 ± 5%) and a daily 12 h light–dark cycle were maintained in the Central Laboratory Animal Facility of the University of Arkansas. All animal handling and experiments were performed in accordance with protocols approved by the Institutional Animal Care and Use Committee of the University of Arkansas (Protocol Approval Number: 17044).

### 2.2. Study Design and Fructose Intervention

IDH2 KO (*idh2*^−/−^ germ-line knockout) mice were bred as we previously described elsewhere [9], and their background strain (C57BL/6N) mice were used as the WT (*idh2^+/+^*) control. For genetic identification of the IDH2 KO mice, tail DNA genotyping was performed. Further, protein and mRNA levels of IDH2 in the liver were also measured. The experimental groups were assigned as follows: WT groups [WT CON and WT Fructose (FRU); *n* = 6 per group] and IDH2 KO groups (IDH2 CON and IDH2 FRU; *n* = 6 per group). WT FRU and IDH2 FRU groups were subjected to 34% fructose solution as described and justified [10] whereas WT CON and IDH2 CON groups were maintained with deionized water. After a six-week intervention, all mice were killed by exsanguination via cardiac puncture under anesthesia, and tissues were harvested and stored at −80 °C for further analyses.

### 2.3. Circulatory Cytokine Array

The Proteome Profiler Mouse Cytokine Array Panel A kit (ARY006; R&D Systems, Minneapolis, MN, USA) was used to monitor serum inflammatory cytokine profiles, which was performed according to the manufacturer’s instructions. Equal volumes (200 μL) of serum and assay buffer (a cocktail of biotinylated detection antibodies) were mixed and then incubated with cytokine pre-coated membranes. The membranes were incubated with HRP-conjugated secondary antibody cocktail, and then the cytokines were detected as dot blots by ChemiDoc™ Imaging Systems (Bio-Rad, Hercules, CA, USA). The dot blots were quantified using ImageJ software (National Institutes of Health, Bethesda, MD, USA).

### 2.4. RNA Isolation and cDNA Synthesis

Stored liver tissues were homogenized and lysed using the QIAzol Lysis Reagent (Qiagen, Hilden, Germany). Total RNA was isolated using the RNeasy Mini Kit (Qiagen) and then the quality of isolated RNA was assessed using the conventional A260/280 ratio and A260/230 ratio measurement (SpectraMax i3x; Molecular Devices, Sunnyvale, CA, USA). The total RNA (2 µg) was then reverse transcribed using the High Capacity cDNA Reverse Transcription kit (Applied Biosystems, Foster City, CA, USA) following the manufacturer’s protocol. The cDNA samples were stored at −80 °C until analyzed.

### 2.5. Quantitative RT-PCR Analysis

Expression of mRNAs was measured by quantitative real time RT-PCR analysis using the StepOnePlus system (Applied Biosystems, Foster City, CA, USA ) in a reaction mixture containing TaqMan Gene Expression Mastermix, primers tagged with TaqMan probe, and cDNA. Amplification was conducted under the following conditions: one cycle at 50 °C for 2 min and 95 °C for 10 min, followed by 40 cycles of denaturation (95 °C for 15 s) and annealing (60 °C for 1 min). Genes of interest were normalized to that of reference genes. Data were analyzed with Step One Software (Ver. 2.1; Applied Biosystems) using the ΔΔCT method. All samples were run in triplicate.

### 2.6. Immunoblot Analysis

Protein expression was assessed using immunoblot blotting. Antibodies for IDH2, actin, NF-κB p65 (p65), NF-κB phosphorylated p65 (p-p65), and interleukin-1β (IL-1β) were purchased from Cell Signaling Technology (Danvers, MA, USA). In brief, protein samples were prepared at a concentration of 1 mg/mL and then dissolved in a 1× sample buffer including 10% sodium dodecyl sulfate, heated for 10 min. Afterwards, samples were separated via sodium dodecyl sulfate polyacrylamide gel electrophoresis and transferred to a nitrocellulose membrane. Membranes were blocked for 1 h in a blocking buffer containing 5% BSA in Tris-buffered saline (0.5 M Tris base, 9% NaCl, and 1% Tween 20; pH 7.8). After blocking, the membranes were incubated with primary antibodies for 12 h at 4 °C. Subsequently, the membranes were washed and incubated with the ScanLater EU-labeled secondary antibodies (Molecular Devices) for 1 h at room temperature. The protein bands were detected using the SpectraMax i3x Multi-Mode Detection Platform (Molecular Devices) and their intensity was quantified using ImageJ software (NIH). Each membrane included a reference sample, which is used in all blots, and the final results were calculated as the ratio of protein/β-actin divided by the ratio of the reference sample/β-actin to factor in interassay variation.

### 2.7. Histological Analyses

Liver tissues were fixed in 4% paraformaldehyde (PFA) in PBS (*w/v*) and embedded in the Tissue-Tek Optimal Cutting Temperature compound (Sakura Finetek, Torrance, CA, USA). Subsequently, tissue sections (5 μm) were prepared for tissue staining. Oil Red O and Hematoxylin and Eosin (H&E) staining were applied to tissue sections for lipid accumulation and morphological observation, respectively. Stained liver sections were then visualized using a BX50 fluorescence microscope (Olympus, Tokyo, Japan).

### 2.8. Statistical Analysis

All results are expressed as least squares mean (LSM) ± standard error mean (SEM). Protein expression data were expressed as fold change (relative quantity) relative to the WT CON group. Differences between the groups were tested using one-way ANOVA followed by multiple comparisons using the difference matrix of least squares mean (SAS Institute, Cary, NC, USA). A *p*-value less than 0.05 was considered statistically different.

## 3. Results and Discussion

**Figure 1:**
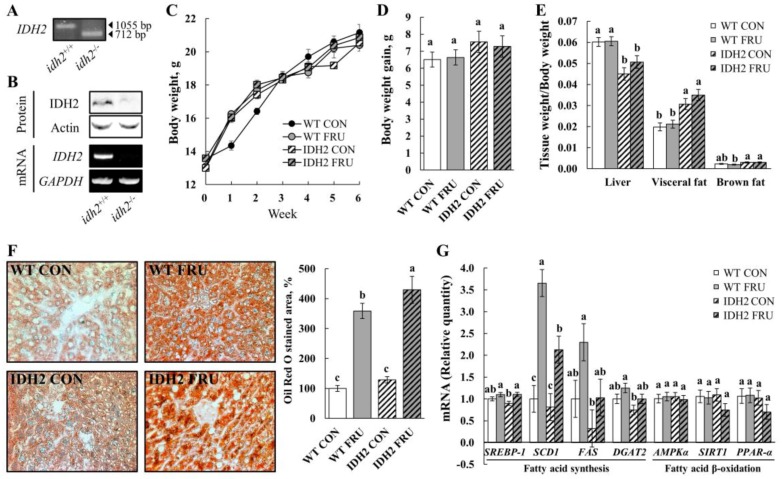
IDH2 knockout aggravates fructose-induced NAFLD conditions. (**A**) IDH2 genotyping was performed using mouse tail DNA; (**B**) protein and mRNA expression of IDH2 in liver tissue of mice were measured by Western blotting and PCR analyses; (**C**) body weight was recorded once a week for six weeks; (**D**) body weight gain was calculated as follows: Final body weight (at sixth week)—Initial body weight (at 0th week). (**E**) Tissue weights of liver, visceral adipose tissue, and brown adipose tissue were represented as the ratio of tissue weights to final body weights; (**F**) hepatic lipid accumulation was assessed by Oil Red O staining, and stained area was calculated using ImageJ software (NIH); (**G**) quantitative PCR analysis of mRNA expressions involved in fatty acid synthesis and β-oxidation (i.e., *SREBP-1*, *SCD1*, *FAS*, *DGAT2*, *AMPKα*, *SIRT1*, and *PPAR-α*) in liver tissue. All data are presented as the LSM ± SEM and different letters indicate statistically significant at *p* < 0.05.

Fructose is added to a variety of processed foods, and its increased intake over the years may be one of the major causes of obesity prevalence and diverse metabolic syndromes including NAFLD [11]. Of note, after 10 years of follow-up, it was recently reported that women are more susceptible to develop metabolic syndromes in response to fructose-sweetened drink consumption [8]. Therefore, we aimed to investigate the effects of fructose on NAFLD conditions in WT and IDH2 KO, specifically in female mice. First, the IDH2 KO mice were genotyped using tail DNA for their genetic identification (Figure 1A) so that we were able to colonize inbred homozygous IDH2 KO female mice. For further confirmation, levels of mRNA and protein were also measured. As expected, neither IDH2 protein nor mRNA was expressed in the liver (Figure 1B).

As the mice accessed food and water ad libitum, fructose-fed mice (WT FRU and IDH2 FRU) had a lower diet intake but consumed a higher amount of fructose-supplemented water. When the amount of food and water (containing fructose) was converted into calories, FRU groups, not counting IDH2 KO or WT mice, consumed more calories than the respective control groups, which is expected given the well-known effects of fructose on calorie intake (WT CON vs. WT FRU; 14.2 ± 0.94 kcal/d/animal vs. 29.8 ± 0.97 kcal/d/animal and IDH2 CON vs. IDH2 FRU; 17.2 ± 4.21 kcal/d/animal vs. 23.7 ± 2.84 kcal/d/animal, *p* < 0.05 for both). However, there was no difference between WT FRU and IDH2 FRU (*p* > 0.05); since a main purpose of this study was to compare responses to fructose between WT and IDH2 KO (neither WT CON vs. WT FRU nor IDH2 CON vs. IDH2 FRU), we believe that this study design is adequate to interpret our results. Despite no difference in body weight and weight gain throughout the study period (Figure 1C,D, respectively; *p* > 0.05), we found visceral fat masses were significantly larger in IDH2 KO groups (Figure 1E). Interestingly, the ratio of liver weight to body weight was significantly decreased in the IDH2 groups compared to the WT groups; not surprisingly, a loss of liver mass is closely related to liver damage and is often followed by inflammatory responses [12], suggesting that IDH2 KO itself may have influenced NAFLD conditions.

In order to further confirm NAFLD conditions, lipid accumulation was assessed by Oil Red O staining of liver tissue sections. As shown in Figure 1F, fructose intervention dramatically increased hepatic lipid accumulation and was even more significantly increased in IDH2 FRU mice (Figure 1F). Expression of key fatty acids synthesis genes was measured to understand how IDH2 KO and/or fructose intervention affects hepatic fatty acid synthesis: *sterol regulatory element-binding protein-1* (*SREBP-1*), *stearoyl-CoA desatuase 1* (*SCD1*), *fatty acid synthase* (*FAS*), and *diacylglycerol O-acyltransferase 2* (*DGAT2*). In this, fructose intervention either induced or presented a trend of increase of all genes’ expression with varying magnitude (Figure 1G), which is consistent with the calorie intake results. It is considered that the difference in calorie intake is a major driver of the altered lipogenic gene expressions in liver tissues. However, our phenotype results (i.e., visceral fat mass, and hepatic fat accumulation) were against the trend of calorie intake, hence gene expressions of *AMP-activated protein kinase α* (*AMPKα*), *sirtuin 1* (*SIRT1*), and *peroxisome proliferator-activated receptor-α* (*PPAR-α*) were assessed to further explore potential mechanisms underlying the phenotypes. There was a decreasing trend in both SIRT1 and PPAR-α expression levels in the IDH2 FRU mice compared to the other groups, which is also in agreement with phenotypes (i.e., Figure 1E). It is generally accepted that PPAR-α is a downstream nuclear receptor of SIRT1; in the liver, SIRT1 positively regulates PPAR-α expression and, in turn, induces PPAR-α targets to increase fatty acid β-oxidation [13]. In our study, the IDH2 KO mice fed fructose showed trends of decreases in SIRT1 and PPAR-α, hinting that fatty acid β-oxidation might have been modulated, warranting additional studies with larger sample size to secure more statistical power.

**Figure 2:**
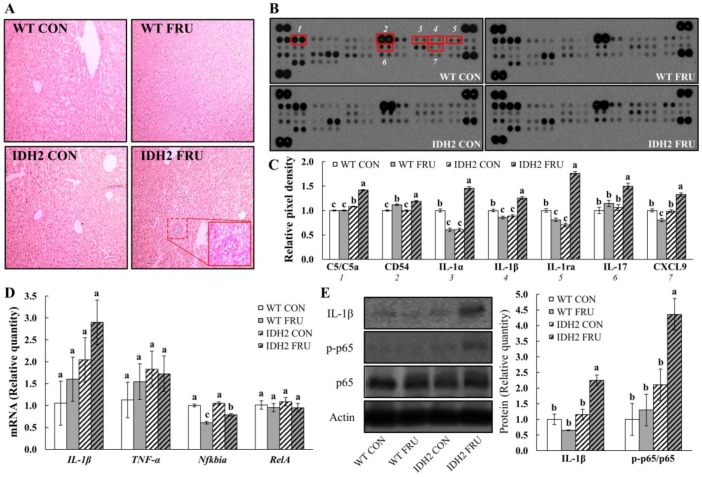
IDH2 knockout exacerbates inflammatory responses via hepatic NF-κB pathway in mice fed 34% fructose over six weeks. (**A**) Morphology of liver tissues was assessed by hematoxylin and eosin staining; (**B**) inflammatory serum cytokines were analyzed using the Proteome Profiler Mouse Cytokine Array Panel A kit (R&D Systems). Levels of 40 cytokines were compared between the experimental groups. (**C**) A graph depicting the relative pixel density of selected dot blots out of the 40 cytokines; (**D**) quantitative PCR analysis of inflammatory mRNA expressions (i.e., *IL-1β*, *TNF-α*, *Nfkbia*, and *RelA*) in liver tissues; (**E**) immunoblot analysis of NF-κB p65 and IL-1β protein expression in liver tissue. Actin was examined as the loading control, and a graph depicting the quantification of the relative abundance of the proteins is shown. All data are presented as the LSM ± SEM and different letters indicate statistically significant at *p* < 0.05.

Subsequently, liver tissue sections were stained by the H&E staining method to see whether there was any physical damage to liver tissues. The H&E staining showed no noticeable indications of steatohepatitis in either the WT group or IDH2 CON groups. However, we observed neutrophil infiltration only in the IDH2 FRU group (highlighted in Figure 2A). Inflammatory signaling pathways play critical roles in NAFLD as ectopic accumulation of hepatic lipids makes liver cells prone to cellular oxidative stress, thereby triggering hepatic inflammation [14]. Thus, we further comprehensively examined circulatory levels of 40 different cytokines in serum samples (Figure 2B). Among the 40 cytokines, seven cytokines were differentially expressed: C5/C5a, CD54, IL-1α, IL-1 β, IL-1ra, IL-17, and CXCL9 (Figure 2C). Interestingly, all seven cytokines were highest in the IDH2 FRU group (*p* < 0.05 for all; Figure 2C); of those, six cytokines were either interleukins or closely related to interleukin signaling. For example, C5/C5a, a strong inflammatory mediator releasing proinflammatory molecule, promotes IL-22 and IL-17 expression in human T cells [15]. In addition, CD54 is known to be induced by IL-1 [16].

To validate the array results, we assessed mRNA expressions of *IL-1β* and its downstream cytokines. There was no significant difference in mRNA expression of *IL-1β*, *TNF-α*, and *RelA* (NF-κB p65 subunit) (Figure 2D). Interestingly, it was noted that *Nfkbia* (NF-κB inhibitor, alpha; IκBα) was significantly downregulated in both WT FRU and IDH2 FRU mice (Figure 2D). Although there was no statistical significance in *IL-1β* level, *IL-1β* is likely to be influenced by both fructose and IDH2 KO (Figure 2D). In addition, considering the fact that *Nfkbia* is an IκBα protein coding gene, a decrease in *Nfkbia* expression is noteworthy. It has been well established that the canonical NF-κB pathway is initiated via the activation of IKK complex; upon activation, phosphorylation-mediated IκBα (coded in *Nfkbia*) elimination occurs, thereby activating the NF-κB cascade [17]. As we observed the increasing trends of *IL-1β* and decreased *Nfkbia* expression by fructose in both WT and IDH2 KO mice, we measured the protein levels of IL-1β and NF-κB p65. Although there is no difference in IL-1β protein expression between the WT CON, WT FRU, and IDH2 CON groups, fructose intervention nearly doubled the hepatic IL-1β in IDH2 KO mice compared to the counterpart (Figure 2E), which is consistent with our cytokine array results and H&E staining phenotype. Since IL-1β is directly related with transactivation of NF-κB p65 [18], we further measured the phosphorylation of NF-κB p65. As expected, the activation of NF-κB p65 was significantly increased in the IDH2 FRU group (Figure 2E). Therefore, our data suggest that fructose reduced the expression of its downstream target (i.e., IκBα (*Nfkbia*)), thereby increasing phosphorylation of NF-κB p65 in IDH2 FRU mice. Consistent with our findings, multiple studies showed that dietary fructose causes NAFLD via activating hepatic inflammatory signaling pathways [19,20]. However, as downregulation of *Nfkbia* was observed in both WT FRU and IDH FRU groups, other mechanisms might have been involved, in addition to this upstream regulator, with regard to the significant increase in phosphorylation of p65 in the IDH2 FRU group.4. Conclusions

For the first time, we demonstrated that IDH2 KO significantly aggravates fructose-induced NAFLD phenotypes and related downstream inflammatory responses in female mice. Further investigations are clearly warranted. especially with regards to the implications of IDH2 in fructose-induced NAFLD development through (1) comprehensive analyses to capture both canonical and non-canonical NF-κB signaling pathways, (2) monitoring the alteration of the metabolites profile in glycolysis and fatty acid synthesis caused by IDH2 deficiency, and (3) elucidating the mechanisms by which IDH2 and fructose intake elicit NAFLD phenotypes in female, but not male mice (see Supplementary Materials). Considering the roles of IDH2 in NADPH production, it is also possible that fructose intervention exacerbated NAFLD development via indirect impairment of the antioxidant defense system as well. Therefore, given our significant phenotypes in response to fructose intervention, the present study provides a good justification for further characterization. Taken together, this study shows that IDH2 deficiency may have implications on fructose-induced NAFLD through modulating fatty acid metabolism and/or activation of the NF-κB pathway.

## Supplementary Materials

The following are available online at http://www.mdpi.com/2072-6643/10/6/679/s1, Figure S1: Effects of fructose supplementation on IDH2 KO male mice.
