# Supplementary material for: IDH2 Deficiency Aggravates Fructose-Induced NAFLD by Modulating Hepatic Fatty Acid Metabolism and Activating Inflammatory Signaling in Female Mice
Source: Nutrients. 2018 May 27;10(6):679. doi: 10.3390/nu10060679 (PMC6024877; doi:10.3390/nu10060679)
Supplement: Supplementary file 1 [file nutrients-10-00679-s001.pdf]

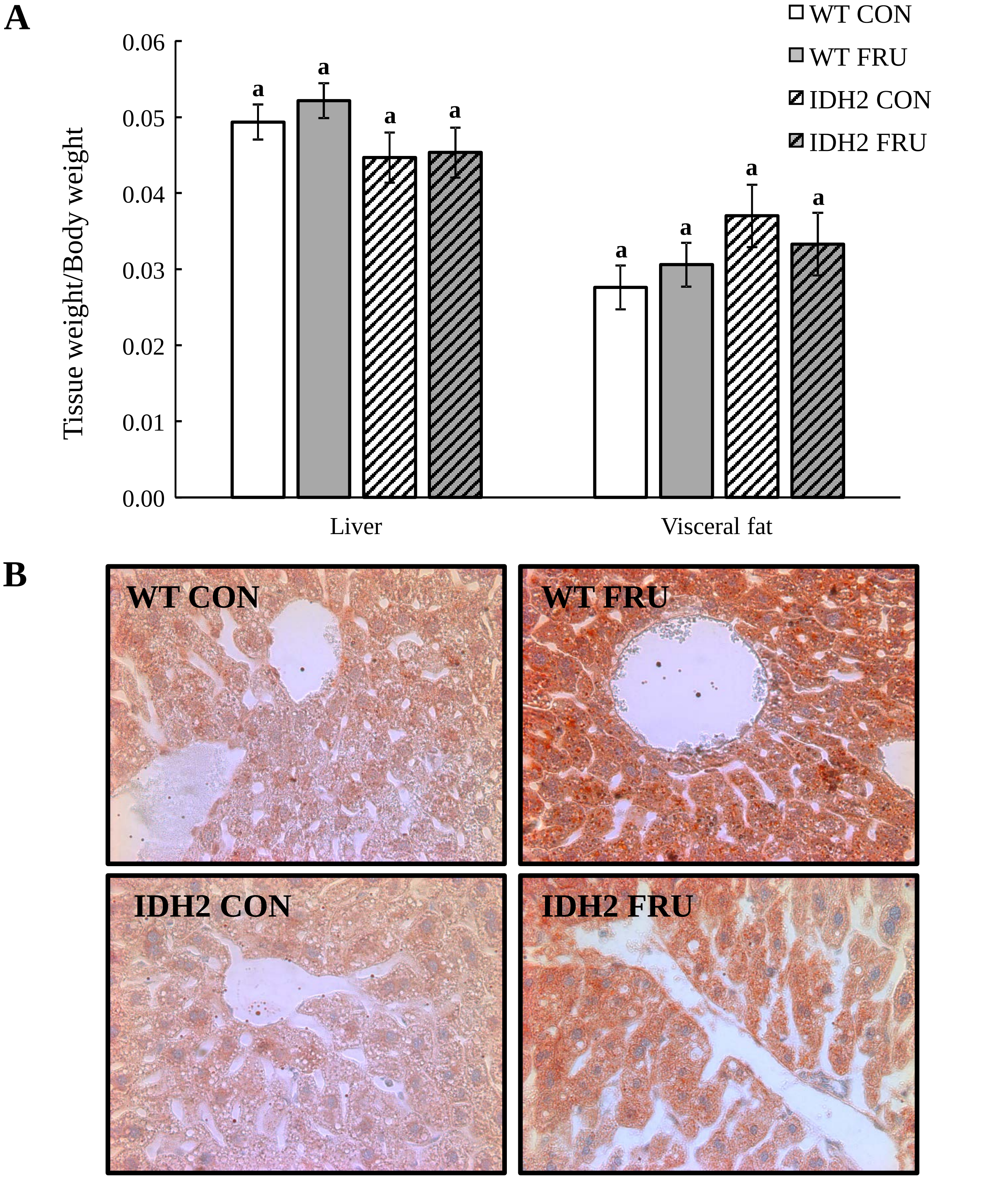

Supplementary material 1. Tissue weights of liver, and visceral adipose tissue in male mice were represented as the ratio of tissue weights to final body weight (A). Hepatic lipid accumulation of male mice were assessed by Oil Red O staining (B). All data are presented as the LSM ± SEM and $P<0.05$ was considered statistically significant.
